# “They’re younger… it’s harder.” Primary providers’ perspectives on hypertension management in young adults: a multicenter qualitative study

**DOI:** 10.1186/s13104-016-2332-8

**Published:** 2017-01-03

**Authors:** Heather M. Johnson, Ryan C. Warner, Christie M. Bartels, Jamie N. LaMantia

**Affiliations:** 1 Division of Cardiovascular Medicine, Department of Medicine, University of Wisconsin School of Medicine and Public Health, H4/512 CSC, MC 3248, 600 Highland Avenue, Madison, WI 53792 USA; 2 Health Innovation Program, University of Wisconsin School of Medicine and Public Health, 800 University Bay Drive, Suite 210, Box 9445, Madison, WI 53705 USA; 3 Department of Counselor Education and Counseling Psychology, Marquette University, Schroeder Health & Education Complex, 561 N 15th Street, Room 151A, Milwaukee, WI 53233 USA; 4 Division of Rheumatology, Department of Medicine, University of Wisconsin School of Medicine and Public Health, 4132 MFCB, Mail Code 2281, 1685 Highland Avenue, Madison, WI 53705 USA

**Keywords:** Qualitative research, Hypertension, Ambulatory care, Health behavior, Medication adherence, Primary healthcare

## Abstract

**Background:**

Young adults (18–39 year-olds) have the lowest hypertension control rates among adults with hypertension in the United States. Unique barriers to hypertension management in young adults with primary care access compared to older adults have not been evaluated. Understanding these differences will inform the development of hypertension interventions tailored to young adults. The goals of this multicenter study were to explore primary care providers’ perspectives on barriers to diagnosing, treating, and controlling hypertension among young adults with regular primary care.

**Methods:**

Primary care providers (physicians and advanced practice providers) actively managing young adults with uncontrolled hypertension were recruited by the Wisconsin Research & Education Network (WREN), a statewide practice-based research network. Semi-structured qualitative interviews were conducted in three diverse Midwestern clinical practices (academic, rural, and urban clinics) using a semi-structured interview guide, and content analysis was performed.

**Results:**

Primary care providers identified unique barriers across standard hypertension healthcare delivery practices for young adults. Altered self-identity, greater blood pressure variability, and unintended consequences of medication initiation were critical hypertension control barriers among young adults. Gender differences among young adults were also noted as barriers to hypertension follow-up and antihypertensive medication initiation.

**Conclusions:**

Tailored interventions addressing the unique barriers of young adults are needed to improve population hypertension control. Augmenting traditional clinic structure to support the “health identity” of young adults and self-management skills are promising next steps to improve hypertension healthcare delivery.

**Electronic supplementary material:**

The online version of this article (doi:10.1186/s13104-016-2332-8) contains supplementary material, which is available to authorized users.

## Background

Hypertension is the most common reversible risk factor for cardiovascular disease [1]. Approximately 20% of young adults (18–39 year-olds) have hypertension and increased risk of heart failure, stroke, and chronic kidney disease [2–5]. Hypertension control reduces morbidity [6, 7] and healthcare costs [8] in young adults. Yet, only 36% of young adults with hypertension have it controlled, compared to 58% of middle-aged and 54% of older adults [5]. Sources of such a wide gap in hypertension control rates between young and mid-to-older adults are not well understood. In fact, once antihypertensive medication is initiated, young adults have higher control rates (70%) than mid-to-older adults [9].

Despite notably poor hypertension control rates, hypertension remains an under-recognized cardiovascular disease risk factor for young adults. Our previous research demonstrated significant delays for young adults to receive an initial hypertension diagnosis [10]. Although lifestyle modifications is important therapy for all young adults with hypertension [11, 12], we demonstrated low rates of lifestyle counseling for young adults with incident (new) hypertension [13]. Additionally, among young adults with severe hypertension (stage 2: ≥160/100 mmHg), we documented significant delays (years) in providers combining lifestyle modification counseling [13] with an initial antihypertensive medication [14]. Multiple studies have also assessed barriers to hypertension control [15–18]. However, understanding the barriers specific to young adults is an important next step to develop effective hypertension interventions. Therefore, we conducted a multicenter qualitative study of primary care providers (physicians and advanced practice providers) caring for young adults with hypertension to assess their recognized barriers to hypertension diagnosis, treatment (lifestyle and medication), and control specific to this population. A separate manuscript focuses on qualitative data from young adults with hypertension [19].

## Methods

### Provider interviews

This study was approved by the University of Wisconsin-Madison Health Sciences Institutional Review Board. One-on-one, 60-min provider interviews were conducted at three Family Medicine/Family Practice clinics within three different counties in Wisconsin, including an academic community clinic, urban clinic, and a rural clinic. One-on-one interviews, instead of focus groups, was the selected approach to provide an environment for physicians to freely share personal information about their practice patterns, identified practice barriers, and knowledge gaps on hypertension clinical care for young adults. Our goal was to promote representation from healthcare providers across diverse practice settings and geographic locations. Providers were recruited by the Wisconsin Research & Education Network (WREN), a statewide practice-based research network, and invited to participate via email and at clinical staff meetings. Purposive non-random sampling was performed with predefined criteria [20]. Inclusion criteria included practicing physicians [medical doctor (MD) or doctor of osteopathic medicine (DO)] and advanced practice providers (physician assistants, nurse practitioners, advanced practice nurse practitioners) with a clinic panel that includes young adults (18–39 year-olds) with uncontrolled hypertension. Across all sites, advanced practice providers were included in the study because they shared patient panels with the physicians. It is common across sites that many patients have follow-up visits with the advanced practice provider. The rural clinical site was the only Family Medicine/Family Practice clinic in the county; the urban and academic clinical sites were the largest ambulatory healthcare delivery systems in their respective counties and were typical of hypertension clinical care in the area. All interviews were conducted by a trained research assistant at each provider’s clinic in a closed office to maintain privacy. Prior to starting the interview, all providers reviewed an IRB-approved summary sheet about the research study and provided verbal consent; written consent (signature) was waived by the University of Wisconsin-Madison Health Sciences Institutional Review Board. All interviews were audio recorded, professionally transcribed verbatim, and reviewed for accuracy. Providers received a $100 honorarium for participation. The data was collected between May 2014 and October 2014.

A semi-structured interview guide was developed based on previous literature of barriers to hypertension control across populations [10, 14, 15, 17, 21–24] and barriers to managing other cardiovascular risk factors among adolescents/young adults (e.g., diabetes [25, 26]). Provider participants were asked a total of 16 questions on the following topics: (1) their personal blood pressure threshold to diagnose hypertension and start lifestyle modification and/or antihypertensive medication, (2) reluctance or hesitancy among themselves or their colleagues to diagnose hypertension and/or start medication, and (3) hypertension guideline applicability for young adults compared to middle-aged and older adults. Samples questions are provided in Table 1.

**Table 1:** Topic guide and sample primary care provider interview questions

Guideline applicability	In your experience, are the JNC (Joint National Committee) 8 guidelines applicable to young adults?
Hypertension diagnosis	In a young adult with multiple elevated blood pressures, is there a point you might consider a diagnosis of hypertension? Would your thoughts and plans for a hypertension diagnosis differ if the patient was older, for example, 55 years old?
Hypertension treatment	If a young adult has elevated blood pressures on multiple visits, is there a point you might consider starting a blood pressure medication? Would your thoughts and plans for starting blood pressure medication differ if the patient was 55 years old?

There were a total of 16 interview questions

### Qualitative data analysis

Our purposive sample size was determined on the basis of theoretical saturation [20]. Data analysis and collection occurred iteratively with adjustment in question content to allow for additional probing [27, 28]. Directed content analysis was used to code the interview transcripts [29]. Initially, transcripts were read to achieve immersion and context. All codes were then determined from the transcribed text, rather than being generated a priori (see Additional file 1). Two investigators without prior clinical hypertension experience (RW and JL) independently coded all transcripts. Emergent codes were generated in initial readings of the transcripts by each coder. The coders then met bimonthly for a multidisciplinary review to adjudicate differences by consensus and refine codes. When the final coding scheme was generated after completion of all interviews, it was applied to all transcripts by a single coder (JL). Data were managed with Microsoft Excel with written protocols and memos of coding and analysis across transcripts. Provider demographic characteristics (gender, years in practice since completing training, type of practice setting (academic, urban, rural) and degree [medical doctor (MD), doctor of osteopathic medicine (DO), nurse practitioner (NP), physician assistant (PA)]) were obtained from self-report and described as continuous and categorical descriptive variables.

## Results

### Participant characteristics

There were a total of 15 Family Medicine/Family Practice providers across all three clinic sites (11 physicians and 4 nurse practitioners). Physicians assistants were not interviewed; either they did not meet inclusion criteria or did not practice in the selected clinics. A total of 10 providers (8 physicians and 2 nurse practitioners) accepted the invitation; 9 completed interviews (one physician canceled secondary to an urgent medical condition). The nurse practitioners and physicians reported similar responses. Table 2 demonstrates that five of the ten providers were female (2 nurse practitioners, 3 medical doctors) which is typical of practice across clinical sites. All interviewed providers reported being of White race and practiced in predominantly White communities. The mean (standard deviation) years in medical practice, after completing training, was 14.4 (±10.2) years.

**Table 2:** Family practice/family medicine primary care provider characteristics

Provider gender	Title	Clinic practice setting	Number of years in clinical practice after training completion
M	MD	Large Multi-Specialty Academic Clinic	12
M	MD		21
F	APNP		23
M	MD	Rural Community Clinic	7
M	MD		34
F	MD		14
F	MD	Urban Community Clinic	<1 year (0.7 months)
F	MD		13
F	NP		5

*MD* Medical Doctor, *APNP* Advanced Practice Nurse Practitioner, *NP* Nurse Practitioner

### Guidelines and young adults

All providers felt that the Joint National Committee 8 (JNC 8) guidelines were applicable to young adults the same as middle-aged and older adults. Providers’ responses unanimously reflected guideline recommendations that at least 2–3 blood pressures from separate visits are needed to confirm elevated blood pressures [12, 30]. In addition, all providers were in agreement that 3–6 months of lifestyle modifications were needed prior to medication initiation, unless the patient had significantly elevated hypertension (i.e., stage 2, ≥160/100 mmHg).

### Reported spectrum of clinical care and barriers

**Fig. 1:**
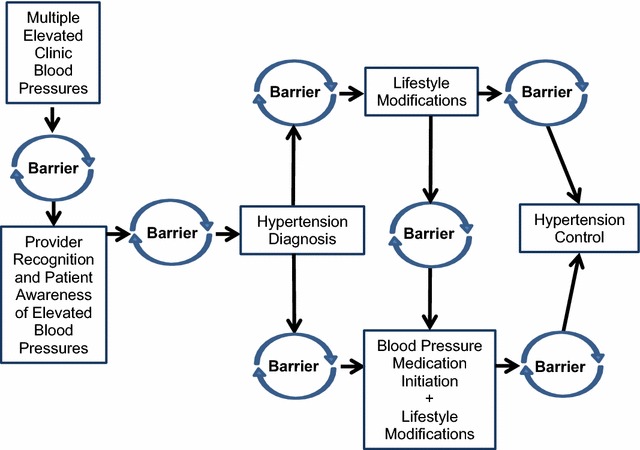
“Barrier points” to deliver hypertension care to young adults. This figure summarizes the most common emergent themes during the 60-min one-on-one Family Practice/Family Medicine provider interviews about barriers and challenges to diagnosis, treat, and control hypertension in young adults

Figure 1 summarizes clinical care transition points that were identified as barriers among the interviewed primary care providers. Compared to middle-aged and older adults, all providers highlighted greater challenges transitioning young adults to the next stage of hypertension management. This most frequently included going from (a) observed blood pressure elevations to an initial hypertension diagnosis and (b) lifestyle modifications alone to lifestyle modifications with blood pressure medication.

### Barrier: psychosocial consequence of a hypertension diagnosis

All primary care providers identified one or more psychosocial consequences as a barrier to providing an initial hypertension diagnosis. The most common theme was that a hypertension diagnosis resulted in a “sick identity” for young adults. Usual management of elevated blood pressures requires young adults to: (1) return more frequently to the clinic for blood pressure checks and (2) sit in clinic waiting rooms usually filled with older patients. This promotes a “sick identity” and precipitates young adults’ resistance to achieving hypertension control. Other psychosocial consequences discussed less frequently were anxiety and fear associated with a new hypertension diagnosis.

- Provider 005: “I think the obstacle is they have to come here to this place (clinic), you know, where they do not see themselves in a sick role. And yet you come here and… you know, you’re in the waiting room and these are people that are sick …where old and sick people come. Maybe we should rethink of where they go and what their self-image is.”

Despite this being a critical barrier, none of the providers could provide alternative healthcare delivery strategies to support young adult’s identity when diagnosing hypertension.

### Barrier: a possible false hypertension diagnosis

The majority of primary care providers were concerned about a false hypertension diagnosis and possible future negative implications, such as obtaining life insurance.

- Provider 005: “It still might affect someone who wanted to buy life insurance to protect their family. You put hypertension as a diagnosis and it really isn’t, it really was episodic elevated blood pressures, then there could be a problem. So I don’t jump on the diagnosis right away.”

When transitioning from recognizing elevated blood pressures to documenting an initial hypertension diagnosis, all primary care providers were concerned about greater clinic blood pressure variability among young adults, especially from “reversible” causes (e.g., stress, white coat syndrome, caffeine, alcohol, tobacco use).

- Provider 005: “Could be smoking, could be caffeine, could be drugs, could be stress, could be all kinds of things and maybe we should follow up.”
- Provider 006: “I’ve had young people who had a bunch of energy drinks… before they came in… I mean …is there some reason that this (elevated blood pressure) is happening now that could be reversible basically?”
- Provider 003: “A lot of them (young adults) lie, and don’t always want to tell you the truth and don’t always want to say what might be in their urine drug screen…”

Given concerns about blood pressure variability and false hypertension diagnoses, the lack of out-of-clinic blood pressures was reported as a significant contributor to delays in providing a hypertension diagnosis for young adults. All providers reported more health insurance coverage among young adults secondary to the Affordable Care Act; however, a recurring policy issue was the lack of standard coverage for home blood pressure monitors. Additionally, although 24-h ambulatory blood pressure monitoring is covered by most medical insurance to exclude white coat hypertension, transportation issues and compliance remain as primary barriers for completing the study among young adults.

- Provider 005: “…Why not do something for young adults, you know? You could have this (blood pressure monitor) in your house and we could monitor it. And realistically, how much do you really need to be in the clinic talking to the doctor for just monitoring when monitoring is more important at that age, you know?”

Attention deficit hyperactivity disorder medications and oral contraceptives were commonly mentioned as contributors to elevated blood pressure in young adults. Although adults ≥40 years old are on similar medications [31], our providers felt that these medications may have a greater contribution to elevated blood pressures in young adults and were uncertain about adjusting or discontinuing these medications versus adding blood pressure medication to lower blood pressure. None of the providers were able to report a resource available to guide treatment decision.

- Provider 003: “Well I can think of some young adults and teenagers who are gaining weight… being on classic ADHD meds. And so I see blood pressure moving up to a point where I’m going to have to say, ‘oops’. And that’s a really big issue because… very often we’ve really carved that medicine out for them… since they were a kid. So that’s a tough thing.”
- Provider 004: “Usually postmenopausal women aren’t on, you know—they could be on low-dose estrogen, but usually it’s just the young women worrying about hypertension caused by their birth control pill.”

### Barrier: visit adherence

The majority of our providers reported lower patient visit adherence among young adults (i.e., higher no-show clinic rates) compared to older adults. During interviews, providers reported numerous life stressors, conflicting demands, transportation barriers, and/or more frequent changes in healthcare systems.

- Provider 001: “Right now a lot of the system relies on us telling a patient to schedule a follow-up, ideally what I try to do is schedule follow-ups at the end of it (clinic visit), so it’s in place. But even then, people, you know, cancel. People no-show. You lose track of people. They’ve got things to do.”

However, possible adverse health effects associated with low visit adherence after starting blood pressure medication was reported by approximately one-third of providers. Per guidelines, some blood pressure medications (e.g., diuretics, ace-inhibitors, angiotensin receptor blockers, aldosterone antagonists) require timely follow-up labs, including electrolyte and renal function monitoring. These providers shared concerns about a risk/benefit ratio of starting a blood pressure medication in a population with low visit adherence.

- Provider 008: “Certain medications, we need to monitor their electrolytes. You know, they’ll take medications but they won’t come back in and get their potassium drawn and they have to go the ER for muscle cramps… You always worry about the side effects of any medication that you start somebody on… are they going to come back?”

### Barrier: gender differences in blood pressure management

The large majority of the primary care provider interviews highlighted gender differences for two recurring topics: (1) clinic blood pressure follow-up and (2) pregnancy risks with blood pressure medication initiation. Of the providers that reported gender-related barriers, over one-third reported it was easier to achieve blood pressure follow-up among young women because of visits required for contraception or because they coordinated visits for other household members (e.g., children). However, most providers reported that because young adult women are of child-bearing age, they felt challenged and were more cautious to start blood pressure medication due to pregnancy risks.

- Provider 006: “Women can’t escape me as much as men. They often need to come in for contraception or for other things and so I can kind of check up on them. So it’s easier for me to keep them in the fold… men could go a long time without seeing a doctor.”
- Provider 003: “I struggle with young women, because of risks in getting pregnant. That’s a tough one (starting blood pressure medication).”

### Barrier: reluctance to start blood pressure medication

All of the providers shared that it was “easier” and they were “less hesitant” to provide an initial hypertension diagnosis to young adults compared to starting medication. The majority of the providers reflected that they are hesitant to require young adults to make a commitment to lifelong medication. Reluctance or hesitancy with medications for other chronic conditions was not discussed in this study.

- Provider 002: “…You know, making a diagnosis or labeling somebody is one thing, putting them on medication is another. With older individuals, I think on average, we have people more commonly on medications. So, adding something in isn’t as much of a change as the young healthy person with a single diagnosis and all of a sudden they’re committed to take… the medication once or twice… a day.”
- Provider 001: “Sometimes it feels like you’re just giving in and just medicating, you know, at an early age. Once you sort of go down that path, you feel like, they’re probably going to be on this indefinitely… I think that there’s a reluctance to medicate… because of a person’s age and the idea that they do have that much time ahead of them, even though the flip side of it is there’s all the more rationale to treat them.”

All of the providers also stressed greater difficulty obtaining young adults’ acceptance of starting antihypertensive medication compared to their older adult patients. Additional research is needed to determine if a provider’s hesitancy or reluctance is reflected onto the patient.

- Provider 005: “And I think this is a little more difficult in young adults. … you know, a 60 year-old who is very afraid of having a heart attack and dying and leaving the family with nothing, they’re pretty easy. You get someone who is 25 years old and you say you have hypertension; but I think that there’s that factor of ‘I’m 25 years old. I’m going to live forever.’ So I think there’s an amount of convincing that you need to do in a young adult that you don’t need to do in an older adult.”
- Provider 006: “They’re younger; it’s really harder for them to take it as seriously. So both sticking with kind of a healthy diet and with exercise and with taking their medications, is probably the biggest challenge.”

However, once the clinical decision was made to consider antihypertensive medication, providers were concerned that medication initiation would promote continued unhealthy behaviors.

- Provider 005: “If I start the medication, does that mean that they then disregard all their other factors… So okay, I’ll take a blood pressure pill and then I’m going to you know, eat all the salt I want and eat all the fat and gain weight and sit around and do nothing.”
- Provider 008: “Do they kind of view it as a crutch, you know? Get their blood pressure down in a normal range and then kind of go back to eating whatever they want or not exercising at all.”

### Barriers to hypertension control across age groups

Our providers shared themes that they felt played a greater role in their practice when managing hypertension in young adults compared to older adults: (a) limited clinic time, (b) financial barriers (e.g., clinic visit co-payments and medication costs), and (c) limited support staff.

- Provider 001: “The pressures of, you know, clinic flow… if you’re lucky you have a 20 min visit to address what we know on average is close to three problems per visit. Not to use it as an excuse, but I think it’s challenging to have effective motivational interviews and outcomes with patients when you have probably 5 min.”
- Provider 003: “Cost is another issue. Although we’ve been working around that for years. So we try to work something out for clinic visits. We have quite a few places where we can get $4 meds or $8 meds or $10 meds.”
- Provider 005: “Non-physician providers like nurse practitioners and physician assistants would be quite helpful, you know, to get them (young adults) to come back so we don’t lose people.”

## Discussion

There has been extensive prior research on barriers to hypertension diagnosis, treatment, and control [15–18, 21]. However, young adults continue to have the lowest hypertension control rates in the United States [5]. To our knowledge, this is the first study to describe barriers and challenges associated with diagnosing, treating, and achieving hypertension control in young adults receiving regular primary care. All of the providers felt that current guidelines (JNC 8) were applicable to young adult populations. However, this study identified an important unintended consequence of our traditional approach to hypertension care—projecting a “sick identity” onto young adults with elevated blood pressures. Our current hypertension guidelines and algorithms do not assist providers with this vital issue, highlighting the critical need for additional research and recommendations within this area. A prior qualitative study of patient perceptions emphasized the importance of reviewing wait times and patients’ perspectives of personal respect [32].

Providers’ concern about greater blood pressure variability is supported by previous research and hypertension scientific statements [33, 34]. Home blood pressure monitoring is recommended to guide hypertension treatment, especially for young adults given their blood pressure variability [33, 35, 36]. Blood pressures measured at home average 6–8 mmHg (systolic)/5–6 mmHg (diastolic) lower than clinic values, which can affect providers’ treatment plans [37]. The lack of out-of-clinic blood pressure data was repeatedly reported as a contributor to delays in a hypertension diagnosis and treatment escalation. Unfortunately, providers reported a lack of resources to defray costs of home blood pressure monitors since they are not covered by many health insurance companies. Longstanding research has demonstrated cost savings and fewer physician visits [38] associated with home blood pressure monitoring, and expanding the affordability and options for out-of-clinic monitors should continue to be a health policy focus [39].

Our discussions about differences in managing hypertension in young adults compared to older adults also yielded recurring themes of barriers that have been historically present across age groups [15–18, 21]. However, visit adherence (i.e., clinic visit no-shows) was an important theme, not just for diagnosis and control of hypertension, but also as a *safety* issue when prescribing blood pressure medication to young adults. It may be beneficial to extend hypertension guidelines to address visit adherence and not just medication adherence. Surprisingly, some providers were concerned that medication initiation promoted young adults to resume or increase unhealthy lifestyle behaviors, which resulted in delays of prescribing medication in their practice. This highlights the need for team-based care to support ongoing lifestyle modification counseling, even between visits, to support a combined approach to hypertension control [40].

Notable gender differences were identified in hypertension care for young adults. In general, young women have more opportunities for blood pressure clinic visits by being a “captured audience.” Previous literature has also demonstrated that young men are less likely to utilize healthcare than women [41]. However, antihypertensive medication initiation among young women poses a greater challenge to primary care providers due to pregnancy risks. Ongoing hypertension quality improvement interventions should address these gender-based barriers to increase population hypertension control.

Strengths of this qualitative analysis include a multisite design including academic, rural, and urban healthcare systems. A separate manuscript summarizes young adult patients’ perspectives on hypertension management from the same healthcare systems [19]. One limitation is that all providers interviewed were Midwestern Family Medicine/Family Practice practitioners and all had low rates of Latino patients on their patient panels. Therefore, this data may not encompass other barriers encountered in managing hypertension in other regions or among some minority races/ethnicities. In addition, the providers interviewed represented patients with primary care access and the identified barriers may not encompass other issues associated with young adults without regular primary care access. All providers across the counties/clinical sites were of White race and practiced in predominantly White communities; however, the state is predominantly White. Therefore, our data may not reflect additional barriers experienced by providers and patients of minority race/ethnicity. In addition, socioeconomic status and neighborhood characteristics across practice panels were not assessed.

## Conclusions

Our qualitative analysis highlights important intervention target areas to hypertension control among young adults. Changes to traditional hypertension healthcare delivery to support ongoing blood pressure self-management and the “health identity” of young adults are needed to address their unique barriers.

## Additional file

**Additional file 1.** Diagnosis and treatment thematic categories and sub-categories created for coding, as determined from the transcribed text for each provider interview.

## Abbreviations

WREN: Wisconsin Research & Education Network
JNC: Joint National Committee
